# Correlation of patient‐reported routine assessment of patient index data with clinical measures of disease activity in psoriatic arthritis

**DOI:** 10.1111/1756-185X.14310

**Published:** 2022-03-25

**Authors:** Louise Ward, Michael Oliffe, Barry Kane, Diana Chessman, Donna Meaney, Fiona Briggs, Kathryn Gibson, Les Barnsley, Daniel Sumpton

**Affiliations:** ^1^ Rheumatology Department Concord Hospital Sydney New South Wales Australia; ^2^ Rheumatology Department Liverpool Hospital Liverpool New South Wales Australia; ^3^ University of New South Wales Sydney New South Wales Australia; ^4^ Concord Clinical School University of Sydney Sydney New South Wales Australia; ^5^ Centre for Kidney Research The Children's Hospital Westmead University of Sydney Sydney New South Wales Australia; ^6^ Present address: Rheumatology Department Royal North Shore Hospital Sydney New South Wales Australia

**Keywords:** arthritis, psoriatic, patient‐reported outcome measures, spondyloarthritis, treatment outcome

## Abstract

**Aim:**

A treat‐to‐target strategy is recommended for management of psoriatic arthritis (PsA), although there is lack of agreement regarding the best measure of disease activity to target. Physician assessments included in traditional indices can be complex and time consuming to complete and cannot be readily conducted by telehealth. This study compares the routine assessment of patient index data 3 (RAPID3), an efficient tool comprising patient self‐assessment, with traditional clinician‐led composite measures in the PsA clinic setting.

**Methods:**

Data were collected prospectively from July 2016 to March 2020 in 2 dedicated PsA clinics in Sydney, Australia. A receiver operating characteristic (ROC) curve was created for comparison of RAPID3 score with composite scores minimal disease activity (MDA), very low disease activity (VLDA) and disease activity in psoriatic arthritis (DAPSA) in low disease activity or remission.

**Results:**

Ninety‐three patients had simultaneous collection of RAPID3 and MDA measures. Mean (SD) age was 49.9 (13.5) years, 50.5% were male and 23 (24.7%) had erosive disease at baseline. RAPID3 scores ≤3.2 and ≤2.7 (range 0‐30) had high sensitivity and specificity for VLDA and DAPSA remission respectively, with ROC curve area under the curve (95% CI) of 0.94 (0.91‐0.97) and 0.96 (0.93‐0.99).

**Conclusion:**

RAPID3 has good agreement with physician‐led composite scores of MDA, VLDA and DAPSA, and provides a viable alternative to composite scores. This is particularly helpful in settings that do not allow for clinical examination, for example telehealth.

## 1 INTRODUCTION

Psoriatic arthritis (PsA) is associated with peripheral and axial joint involvement and distinctive extra‐articular manifestations.^1^ It is associated with increased rates of cardiovascular disease, mortality, mental health burden and has a significant impact on quality of life.^2^, ^3^, ^4^, ^5^, ^6^ A treat‐to‐target (T2T) strategy, aiming for early remission or low disease activity, is recommended to reduce symptoms of pain and stiffness, improve joint function and reduce the risk of reversible joint damage.^7^, ^8^ Various composite measures to assess PsA disease activity have been developed and validated, although there is no agreement on the optimal measure.^9^, ^10^, ^11^ Such measures include minimal disease activity (MDA), very low disease activity (VLDA), disease activity index for psoriatic arthritis (DAPSA), Psoriatic Arthritis Disease Activity Score and Composite Psoriatic Disease Activity Index.^12^, ^13^, ^14^, ^15^

Composite measures require multiple clinical and laboratory measures to be recorded, rendering them impractical for busy clinical settings and utilizing time and resources that could be better spent undertaking patient‐centered care activities, such as education and shared discussion of treatment options.^16^, ^17^ Patient‐reported outcome measures (PROMs) offer a potential efficient, patient‐led alternative to composite activity measures. If reliable and accurate, they could increase time available in consultation for discussion regarding care and education, thereby improving quality of care.^18^

The routine assessment of patient index data 3 (RAPID3) is a PROM initially developed in patients in rheumatoid arthritis. It efficiently provides results comparable to validated disease activity scores in clinical trials and clinical practice.^19^, ^20^, ^21^ RAPID3 has been demonstrated to be useful in patients with PsA but has not been extensively studied and there is limited understanding of the expected RAPID3 values to describe low disease activity or disease remission.^22^, ^23^

This study aims to assess the ability of RAPID3 to discriminate disease activity in PsA in the clinical practice setting, as compared to assessment by MDA, VLDA and DAPSA, and to determine the respective optimal RAPID3 cut points that correspond to categories of disease activity, as per the composite measures.

## 2 METHODS

Consecutive patients were recruited from PsA clinics in the rheumatology departments at Concord Repatriation General Hospital and Liverpool Hospital, both tertiary teaching institutions in Sydney, Australia. To be included in the study, participants had to be at least 18 years old, meeting the Classification Criteria for Psoriatic Arthritis (CASPAR) and able to read English. All persons gave their informed consent prior to their inclusion in the study. Participants had to be willing to complete PROMs, be assessed for clinical measures at each visit and have at least 1 simultaneous collection of RAPID3 and MDA/VLDA scores. Patients were excluded if they could not read English, did not meet CASPAR or were unwilling to fill out PROMs or undertake complete clinical assessments. Ethics approval was granted by the South Western Sydney Local Health District Ethics Committee (HREC No: HREC/15/LPOOL/560). Site‐specific approval was granted for Concord Hospital.

Participants were assessed for all clinical measures required for MDA, VLDA and DAPSA criteria, including tender joint count in 68 joints (TJC68), swollen joint count in 66 joints (SJC66), enthesitis count and skin assessment according to the Group for Research and Assessment of Psoriasis and Psoriatic Arthritis (GRAPPA) methods.^24^ Scores were recorded on standardized proformas by appropriately trained physicians. For skin assessments, assessors used Psoriasis Area and Severity Index (PASI) and body surface area (BSA) according to standard protocols.^25^, ^26^

Achieving MDA requires meeting 5/7 criteria: TJC68 ≤1, SJC66 ≤1, PASI ≤1 or BSA ≤3%, patient global activity (PtG) ≤20 on a visual analog scale (VAS; range 0‐100), patient pain (PP) on VAS ≤15 (range 0‐100), Health Assessment Questionnaire (HAQ) ≤0.5 and tender entheseal count ≤1.^27^ If all 7 criteria are met, the patient is in VLDA. DAPSA comprises a sum of TJC68, SJC66, PtG (range 0‐10), PP (range 0‐10), and C‐reactive protein result (mg/dL). The following cut points for levels of disease activity were used: remission ≤4, low disease activity 4.1‐14, moderate disease activity 14.1‐28, high disease activity >28.^28^

RAPID3 is an index composed of physical function, pain, and patient global assessment, each scored 0‐10 for a total of 0‐30 on the Multi‐Dimensional HAQ (MDHAQ).^29^, ^30^, ^31^, ^32^ RAPID3 has demonstrated correlation with composite disease activity measures in rheumatoid arthritis and disease‐specific questionnaires in osteoarthritis, systemic lupus erythematosus, spondyloarthropathy and gout.^29^, ^31^, ^32^, ^33^ Patients were posted PROMs including RAPID3 and HAQ in the week prior to their visit and instructed to fill out the PROMs 24 hours prior to their clinical assessment. If patients had not completed the self‐assessment tools, they were invited to complete them in the waiting room prior to their appointment.

For each clinic visit, we determined the patients' DAPSA score and whether a patient was in MDA or VLDA. The analysis of usefulness of RAPID3 to determine disease activity status was performed using receiver operating characteristic (ROC) methodology and SPSS software. Binary logistic regression analysis was performed for each of the 4 clinician‐led composite measures and compared to every RAPID3 score collected during the study. ROC curve analysis was performed for comparison of the continuous RAPID3 score with binary outcomes of MDA, VLDA, DAPSA remission (DAPSA‐REM) and DAPSA low disease activity (DAPSA‐LDA; ie, DAPSA score ≤14). Area under the curve (AUC) of each ROC curve was calculated and interpreted using the accepted analysis of diagnostic accuracy, with AUC of 0.5 providing no discrimination, 0.8‐0.9 demonstrating excellent accuracy and 1 representing perfect accuracy.^34^ The optimal cut point was selected by choosing the data point closest to (0,1), representing the optimal trade‐off between sensitivity and specificity.

## 3 RESULTS

One hundred and three patients were enrolled, and data were collected from July 2016 to March 2020. Nine patients were excluded due to missing data and 1 patient later withdrew consent to complete required PROMs. Simultaneous RAPID3 and MDA/VLDA data were available for 336 clinic visits from 93 patients (44 and 49 from Concord and Liverpool Hospitals, respectively) and simultaneous RAPID3 and DAPSA data were available for 85 patients over 290 clinic visits. The median (interquartile range) number of visits per patient was 3 (1‐5). At baseline, mean (±SD) age was 49.9 (±13.5) years with 47 (50.5%) male patients (Table 1). Twenty‐three (24.7%) patients had erosive disease. The mean duration of PsA was 10.4 years. Thirty‐three (35.5%) patients were taking methotrexate at baseline and 41 (44.1%) were taking a biologic or targeted synthetic disease‐modifying agent (Table 2).

**TABLE 1:** Demographic data

Variable n (%), mean ± SD or median (IQR) or as specified	Result
Age, y	49.9 ± 13.5
Male	47 (50.5)
Body mass index, kg/m^2^	31.6 ± 7.5
PsA characteristics	
PsA duration, y	10.4 ± 10.4
Axial disease	19 (20.4)
Tender joint count	7.0 (2.0‐13.5)
Swollen joint count	1.0 (0.0‐5.0)
PASI	1.6 (0.3‐3.8)
C‐reactive protein, mg/dL, mean (range)	64.2 (0‐704.0)
DAPSA score	25.2 ± 20.7
RAPID3 score	12.3 (7.3‐17.5)
Comorbidities	
Hypertension	38 (40.1)
Osteoarthritis	25 (26.9)
Diabetes	23 (24.7)
Mental illness	22 (23.7)
Smoking	13 (14.0)

Abbreviations: DAPSA, disease activity in psoriatic arthritis; PASI, Psoriasis Area and Severity Index; PsA, psoriatic arthritis; RAPID3, routine assessment of patient index data 3.

**TABLE 2:** Medications

Medications	n (%)
csDMARDs	
Methotrexate	33 (35.5)
Sulfasalazine	14 (15.1)
Leflunomide	4 (4.3)
Cyclosporin	1 (1.1)
bDMARDs	
Secukinumab	12 (12.9)
Adalimumab	10 (10.8)
Ustekinumab	6 (6.5)
Etanercept	5 (5.4)
Golimumab	4 (4.3)
Infliximab	2 (2.2)
tsDMARD	
Tofacitinib	2 (2.1)
Corticosteroid	
Prednisone	11 (11.8)

Abbreviations: bDMARDs, biological disease‐modifying antirheumatic drugs; csDMARDs, conventional synthetic disease‐modifying antirheumatic drugs; tsDMARD, targeted synthetic disease‐modifying antirheumatic drug.

At baseline, 4 (4.3%) and 17 (18.3%) patients met VLDA and MDA, respectively and 9 (10.6%) and 24 (28.2%) patients met DAPSA‐REM and DAPSA‐LDA, respectively. Over the course of the data collection period, 17 patients met MDA criteria across 81 (24.1%) clinic visits and 12 met VLDA criteria at 24 (7.1%) clinic visits. Of the 290 clinic visits with complete DAPSA data, there were 110 (37.9%) events in which 44 patients met DAPSA‐LDA and 38 (13.1%) visits in which 16 met DAPSA‐REM.

**FIGURE 1:**
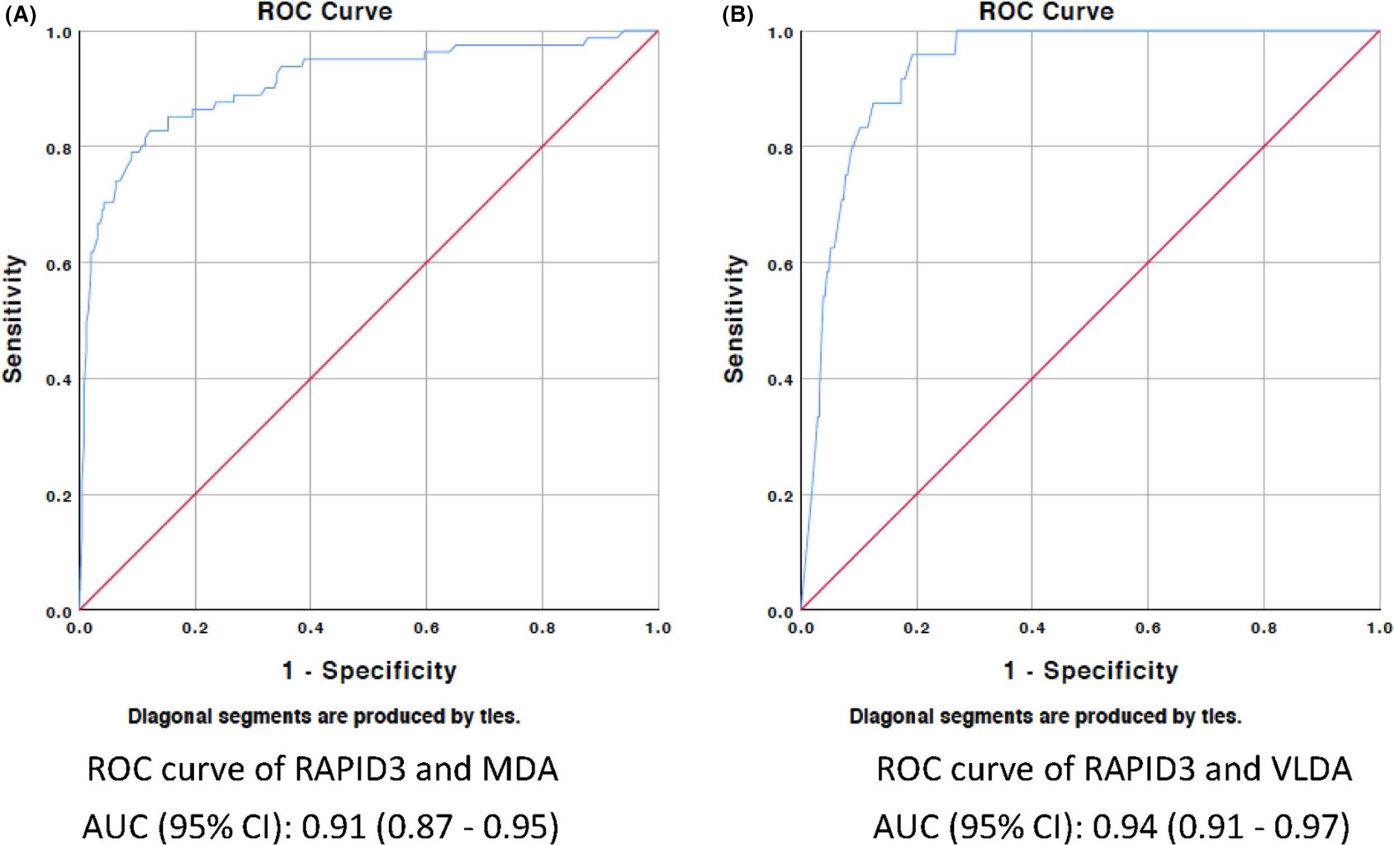
Receiver operator characteristic (ROC) curve for minimal disease activity (MDA)/very low disease activity (VLDA) and routine assessment of patient index (RAPID3). The ROC curves show the ability of RAPID3 to identify patients who meet MDA activity criteria (Figure 1A) and those who meet VLDA criteria (Figure 1B). The red line is a reference line indicating area under the curve (AUC) of 0.5, corresponding to no discriminatory ability of the test. The AUC (95% CI) for RAPID3 to discriminate patients who meet MDA compared to patients who do not meet criteria was 0.91 (0.87‐0.95). The AUC (95% CI) for RAPID3 to discriminate patients who meet VLDA criteria was 0.94 (0.91‐0.97)

**FIGURE 2:**
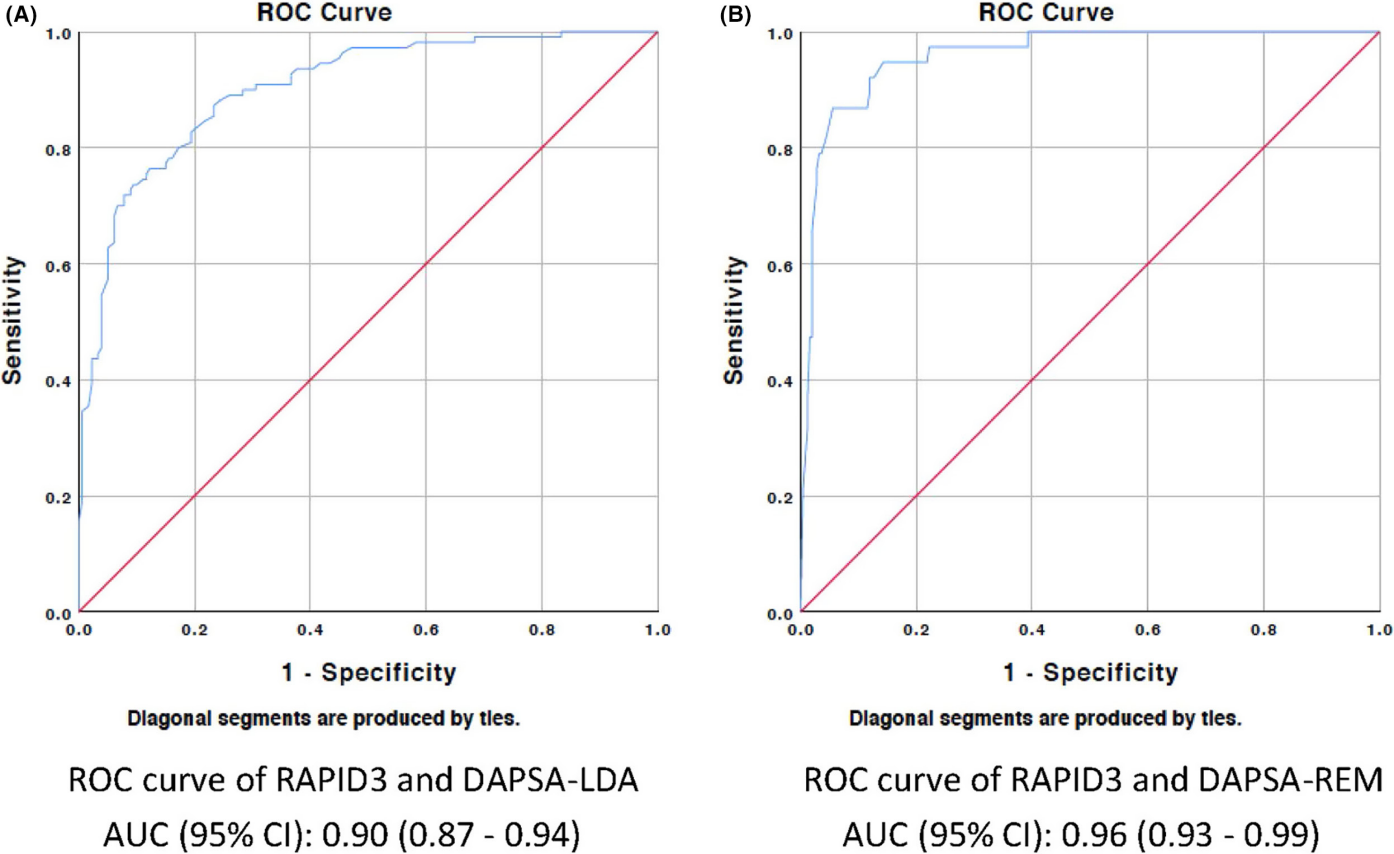
Receiver operator characteristic (ROC) curve for disease activity in psoriatic arthritis in low disease activity and remission (DAPSA‐LDA)/disease activity in psoriatic arthritis in remission (DAPSA‐REM) and routine assessment of patient index (RAPID3). The ROC curves show the ability of RAPID3 to identify patients who meet DAPSA‐LDA (Figure 2A) and DAPSA‐REM criteria (Figure 2B). The red line is a reference line indicating area under the curve (AUC) of 0.5, corresponding to no discriminatory ability of the test. The AUC (95% CI) for RAPID3 to discriminate patients who meet DAPSA‐LDA compared to patients who do not meet criteria was 0.90 (0.87‐0.94). The AUC (95% CI) for RAPID3 to discriminate patients who meet DAPSA‐REM criteria was 0.96 (0.93‐0.99)

Binary logistic regression analysis was performed for each of the 4 composite measures with RAPID3. The AUC of the generated ROC curves are shown in Table 3. The optimal RAPID3 cut points were 6.0, 3.2, 10.0 and 2.7 for MDA, VLDA, DAPSA‐LDA and DAPSA‐REM respectively. The sensitivity and specificity of each of the selected cut points are reported in Table 3. The generated ROC curves are displayed in Figures 1 and 2.

**TABLE 3:** Receiver operating characteristic curve output

	AUC (95% CI)	Optimal RAPID3 cut point	Sensitivity of RAPID 3 cut point (%)	Specificity of RAPID 3 cut point (%)
MDA	0.91 (0.87‐0.95)	6.0	82.7	87.8
VLDA	0.94 (0.91‐0.97)	3.2	87.5	87.5
DAPSA‐LDA	0.90 (0.87‐0.94)	10.0	82.7	80.6
DAPSA‐REM	0.96 (0.93‐0.99)	2.7	86.8	94.0

Abbreviations: AUC, area under the curve; RAPID3, routine assessment of patient index data 3; MDA, minimal disease activity; VLDA, very low disease activity; DAPSA‐LDA, disease activity in psoriatic arthritis – remission/low disease activity; DAPSA‐REM, disease activity in psoriatic arthritis – remission.

**FIGURE 3:**
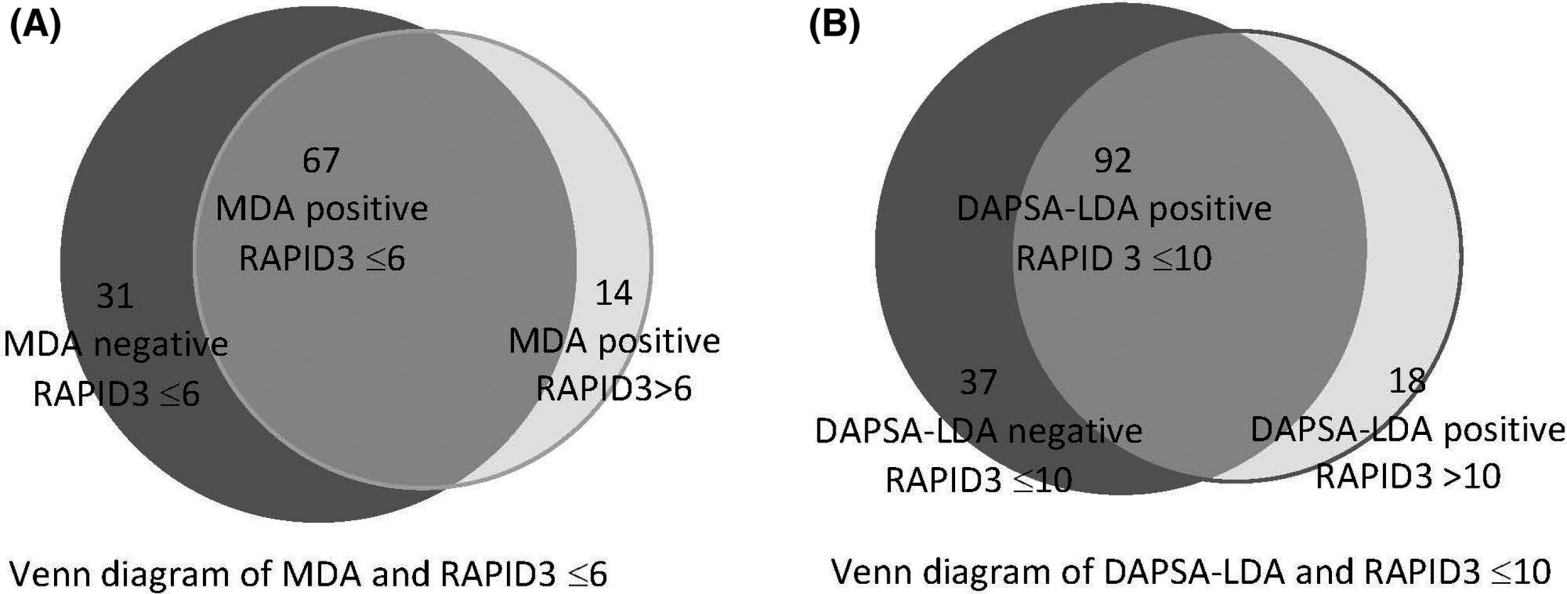
Venn diagram for the relationship of routine assessment of patient index (RAPID3) with minimal disease activity (MDA) and disease activity in psoriatic arthritis with low disease activity (DAPSA‐LDA). The Venn diagram in Figure 3A demonstrates the relationship between the number of patient visits in which patients met MDA criteria (light gray; Figure 3A) and the number of patient visits in which patients reported a RAPID3 score ≤6 (dark gray; Figure 3A). In 67 patient visits, patients were in MDA and scored RAPID3 ≤6. In 14 patient visits, patients were in MDA but scored RAPID3 >6. Figure 3B demonstrated the relationship between patients who meet DAPSA‐LDA criteria (light gray; Figure 3B) and the number of patients who report a RAPID3 score ≤10 (dark gray; Figure 3B). In 92 patient visits, patients were in DAPSA‐LDA and scored RAPID3≤10. In 18 patient visits, patients were in DAPSA‐LDA but scored RAPID3 >10

The relationships between patients in a state of MDA and DAPSA‐LDA and the optimal RAPID3 cut points of 6 and 10 respectively are demonstrated in Figure 3. The majority of patient visits in which patients were in MDA were associated with a RAPID3 score ≤6 (Figure 3A). In 67 patient visits, scores were concordant scores (ie patients both in MDA and achieving RAPID3 score ≤6). In 14 patient visits, patients were in MDA but had a RAPID3 score exceeding 6 and in 31 patient visits, patients had a RAPID3 score ≤6 but patients did not meet MDA criteria. Not depicted in the Venn diagram are the 224 patient visits where patients did not meet MDA and had RAPID3 >6, that is concordant scores for patients with greater than minimal disease activity. Similar results are noted for the relationship between patients with a RAPID3 score ≤6 and in DAPSA‐LDA (Figure 3B).

The distribution of RAPID3 scores for patients in MDA and for patients not in MDA is represented in the supplementary data and demonstrates the ability of RAPID3 scores ≤6 to identify patients who are likely in a state of minimal disease activity (Figure S1). Patients who are not in a state of MDA are more likely to have a RAPID3 score >6 (Figure S2). Figure S3 shows the range of RAPID3 and DAPSA scores across all patient visits and this scatter plot illustrates the linear relationship between RAPID3 scores and DAPSA scores for each individual visit.

## 4 DISCUSSION

In this study, self‐reported RAPID3 scores from patients attending specialty PsA clinics were compared with clinician‐assessed composite disease activity scores. This study demonstrated high discriminatory ability of RAPID3 in assessing disease activity in PsA. The AUC of ROC curve for RAPID3 with each of the 4 selected disease activity measures exceeded 0.9, consistent with excellent diagnostic accuracy. The optimal RAPID3 cut points for VLDA and DAPSA‐REM were 3.2 and 2.7, respectively. The RAPID3 cut point that gave best sensitivity and specificity to meet MDA and DAPSA‐REM were 6.0 and 10.0, respectively.

The high degree of concordance of RAPID3 scores using the above cut points with MDA and DAPSA‐LDA is visualized in the Venn diagrams in Figure 3 and scatter plots in Figures [Link], [Link], [Link].

This study provides a novel understanding of how RAPID3 could be used to estimate disease activity in PsA by defining the cut points for assessments of disease activity based on 2 composite scores. RAPID3 cut points used in RA are ≤3 for remission, 3.1‐6.0 for low disease activity, 6.1‐12 for moderate disease activity and >12 for high disease activity.^20^ In our study, the identified optimal RAPID3 cut points for remission (as defined by VLDA and DAPSA‐REM), approximate the RAPID3 cut point for remission in rheumatoid arthritis (ie, RAPID3 ≤3).

RAPID3 has been shown to correlate with other measures of disease activity in PsA. Coates et al^22^ performed a post hoc analysis on clinical trial data in PsA to compare RAPID3 with PsA disease activity scores. Results demonstrate agreement between MDA and RAPID3 remission in 85.2% of patients and significant correlation between RAPID3 scores and DAPSA criteria. In contrast, Walsh et al^23^ found low correlation between RAPID3 and swollen and tender joint counts, although this clinical measure does not take into account the additional facets of PsA that are considered in composite measures, for example skin and tender entheseal count.

Patient‐reported outcome measures such as RAPID3 present an opportunity for improved patient‐centered care with applications in multiple healthcare models. First, with respect to inclusion of PROMs in the traditional face‐to‐face appointment model of rheumatology care, patients have observed that completion of PROMs prior to a physician consultation can increase the efficiency of the appointment with the rheumatologist.^35^ PROMS have the potential to facilitate shared decision making, guide patient‐physician communication and provide feedback for progress over time.^36^, ^37^ Second, the application of PROMs in settings independent of traditional appointments presents a promising opportunity to empower patients. In a recent pilot study employing online self‐monitoring in patients with inflammatory arthritis, patients reported increased knowledge and awareness of their disease, with some noting earlier self‐recognition of disease flare.^38^ Self‐monitoring presents a potential counter to key challenges identified by patients with PsA and psoriasis, notably fear of deterioration, lack of control and disempowerment by lack of personalized care.^39^

The use of PROMs also presents a feasible and efficient alternate in assessment of disease activity to facilitate the T2T model. Despite the strong evidence for its use, T2T has low uptake in the clinical setting due to various barriers including time limitations and need for training.^17^ The application of PROMs electronically, as discussed above, presents an opportunity for remote disease assessment to be incorporated in the T2T model.^40^ The use of remote PROMs in assessment of disease activity has never been more compelling, given the current limitations in face‐to‐face appointments in the setting of the COVID‐19 pandemic. Our study supports the use of RAPID3 to estimate disease activity to enable adjustment of therapy to target remission. RAPID3 is a feasible choice of PROM in the busy clinical setting. It is non‐proprietary for clinical use, takes 5–10 seconds to complete and does not require extra clinic time or physician training.^30^ It was designed for use in clinical practice, as opposed to use in the clinical trial setting and can be easily electronically administered.^37^

While RAPID3 was not specifically designed for use in PsA and therefore lacks the specific PsA clinical manifestations of psoriasis, enthesitis and axial disease, it has the advantage of established validation in a wide range of rheumatological disorders, thus allowing comparison.^41^ Further, the addition of a skin VAS has been previously shown to not substantially improve disease activity estimation.^22^ Extending the application of RAPID3 to also include the symptom checklist in the MDHAQ provides opportunity for clinicians to expand screening to include axial symptoms, symptoms suggestive of inflammatory eye and bowel disease, fatigue and stiffness. This may particularly be helpful when assessing patients who are not achieving MDA. Psoriatic Arthritis Impact of Disease (PSAID) is a PROM designed specifically for use in PsA and presents a potential alternative PROM for use in the PsA clinic setting.^42^ It has been demonstrated to correlate strongly with RAPID3 and has been endorsed by Outcome Measures in Rheumatology as a core outcome measure to assess health‐related quality of life in PsA.^23^, ^43^

Data were collected prospectively from the clinical setting from 2 separate centers, including approximately 300 clinic visits for each disease activity measure. All rheumatologists received training to perform clinical assessments to calculate the disease activity measures, but intra‐ or inter‐assessor variability was not formally assessed. Given these data were collected in the clinical setting, there is a high likelihood that the same examiner assessed the patient on their multiple visits, due to a general aim for continuity of care, thereby amplifying potential subjectivity. One rheumatologist assessed patients at both hospitals. Further, examiners were not blinded to RAPID3 results prior to the clinic visit. The exclusion of patients who were unable to read English limits the generalizability of this study. A further limitation of data is the incomplete collection of data for DAPSA and screening for potential confounders, such as fibromyalgia.

Further research is needed to define the role of RAPID3 in the T2T approach. While this prospective study has demonstrated that RAPID3 and composite disease activity measures are highly correlated, we have not determined whether RAPID3 is responsive to treatment or sensitive to change over time with treatment. Qualitative analysis of the patient experience with RAPID3 in the PsA setting would further inform its inclusion into regular clinical care.

In conclusion, RAPID3 is able to discriminate between levels of disease activity in PsA. This study supports the use of a RAPID3 score cut point of ≤3 for remission in PsA. RAPID3 offers a potential solution for assessment of disease activity in situations where clinical assessment is not readily possible, for example with telehealth consultations.

## CONFLICT OF INTEREST

The authors do not have financial interests that could create a potential conflict of interest or the appearance of a conflict of interest with regard to the work.

## Supporting information

Fig S1

Fig S2

Fig S3

Supplementary Material
