# Supplementary figures and images for: Correlation of patient‐reported routine assessment of patient index data with clinical measures of disease activity in psoriatic arthritis
Source: Int J Rheum Dis. 2022 Mar 25;25(5):584–91. doi: 10.1111/1756-185X.14310 (PMC9310573; doi:10.1111/1756-185X.14310)

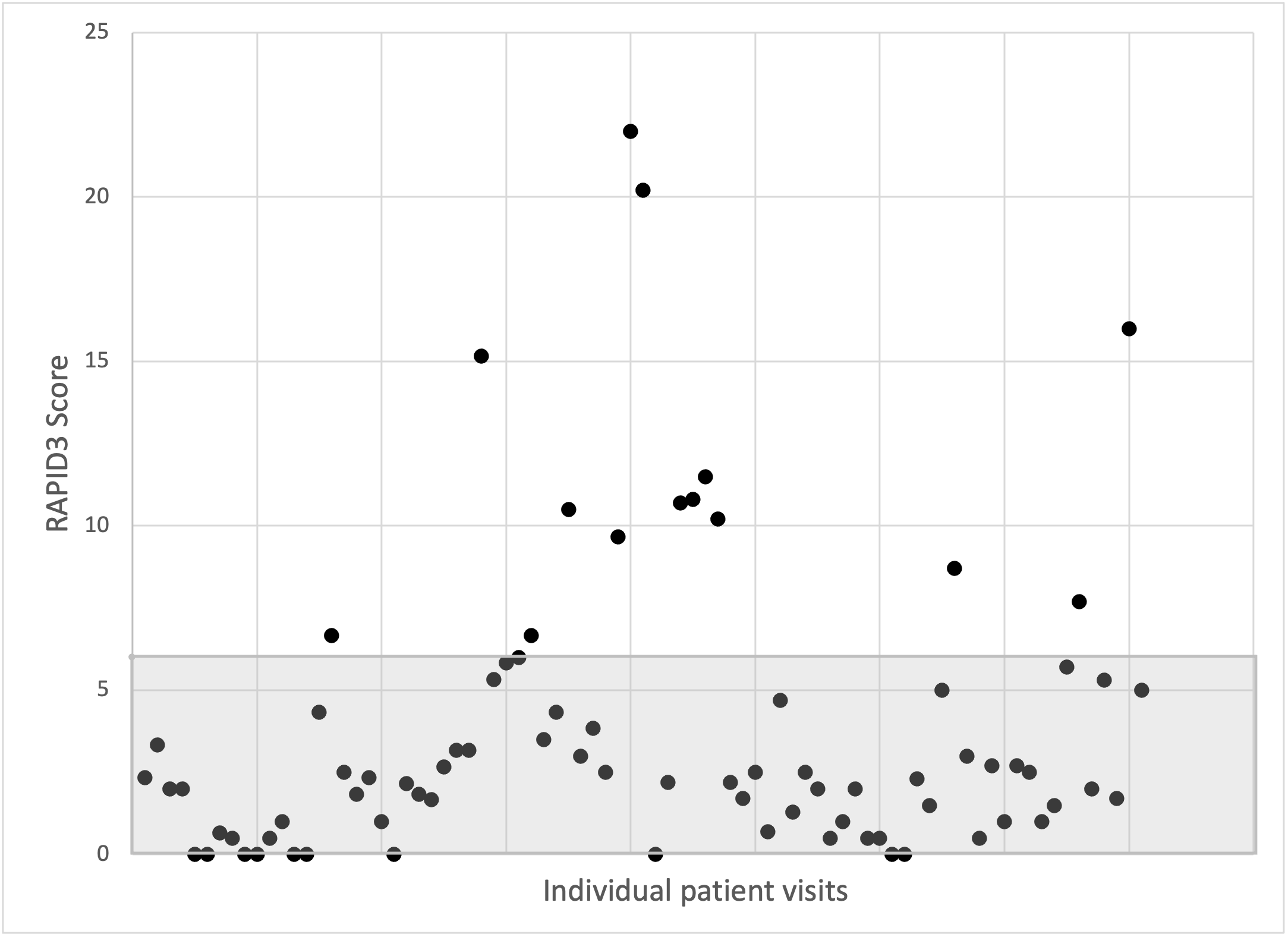

Supplement: Supplementary file 1 — Fig S1 [file APL-25-584-s002.png]

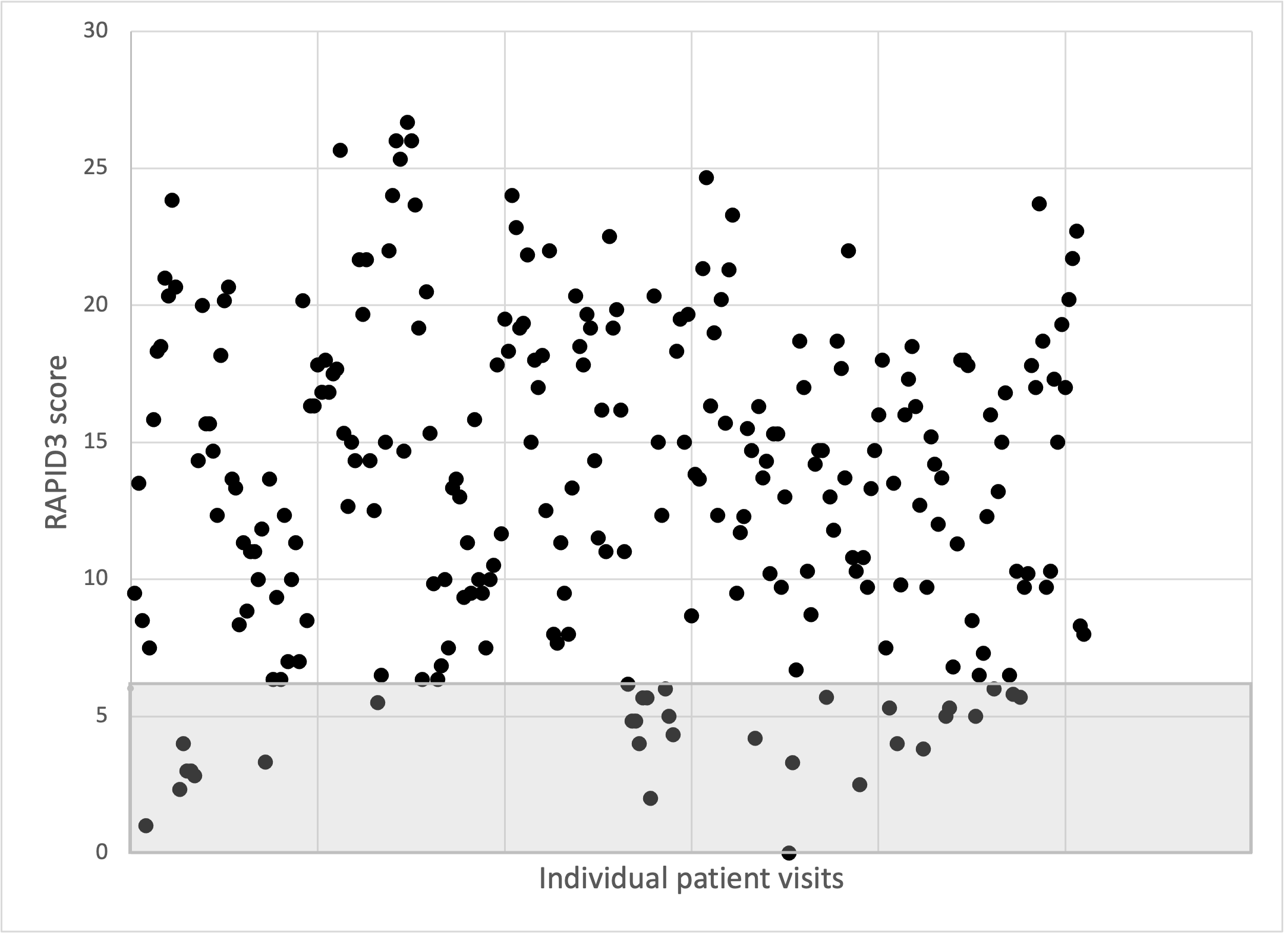

Supplement: Supplementary file 2 — Fig S2 [file APL-25-584-s004.png]

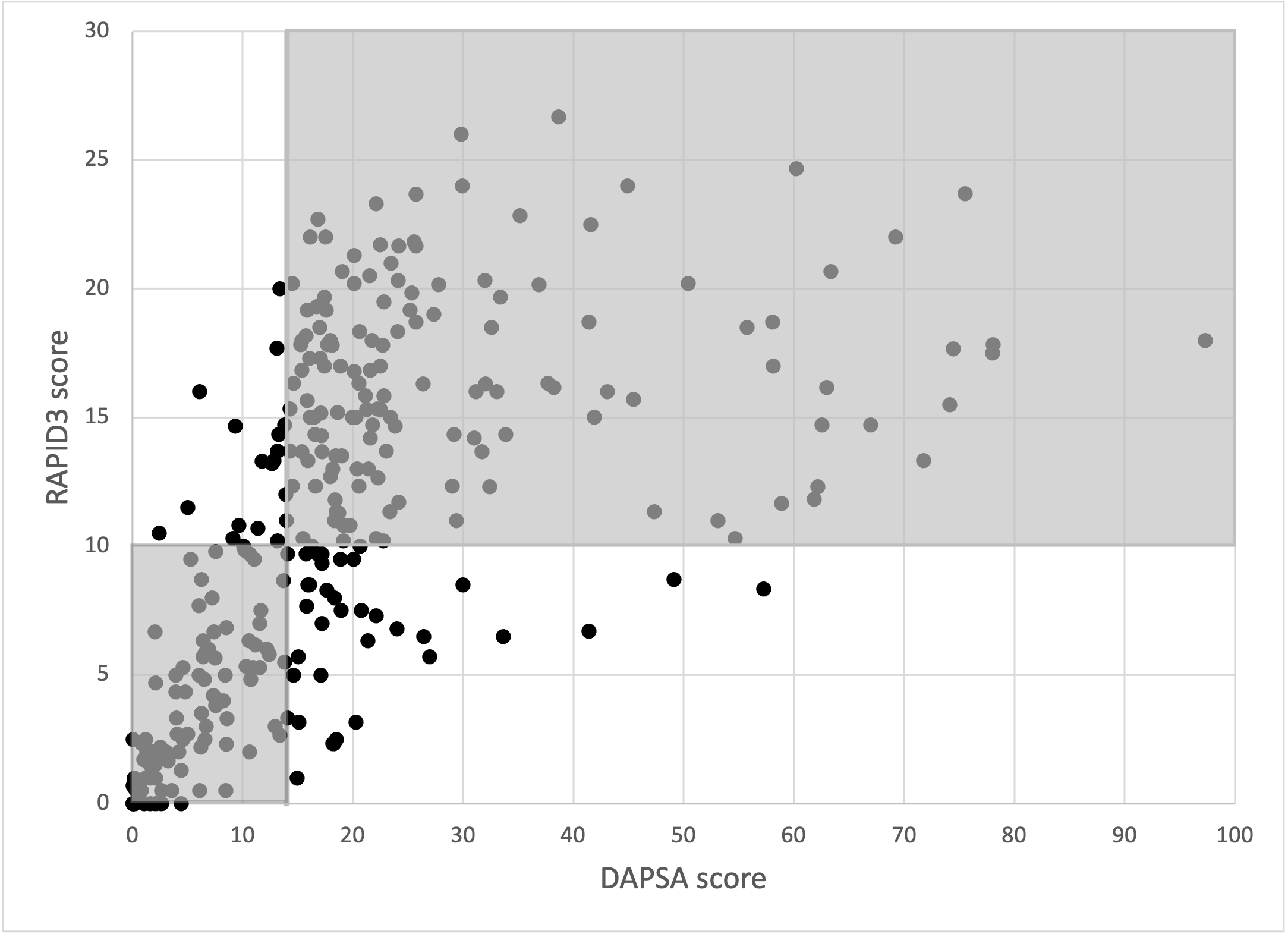

Supplement: Supplementary file 3 — Fig S3 [file APL-25-584-s001.png]
