# Supplementary material for: Correlation of patient‐reported routine assessment of patient index data with clinical measures of disease activity in psoriatic arthritis
Source: Int J Rheum Dis. 2022 Mar 25;25(5):584–91. doi: 10.1111/1756-185X.14310 (PMC9310573; doi:10.1111/1756-185X.14310)
Supplement: Supplementary file 4 — Supplementary Material [file APL-25-584-s003.docx]

**Supplementary Figure 1 Distribution of rapid assessment of patient index (RAPID3) scores in patient visits in which patients achieved minimal disease activity (MDA)**

The scatter plot shows the distribution of RAPID3 scores for visits in which patients achieved MDA. The shaded area represents the range of RAPID3 scores that are ≤6. Therefore, the data points falling within the shaded area are concordant, ie patients achieving MDA and RAPID3 score ≤6.

**Supplementary Figure 2 Distribution of rapid assessment of patient index (RAPID3) scores in patient visits in which patients did not achieve minimal disease activity (MDA)**

The scatter plot shows the distribution of RAPID3 scores for patients not achieving MDA. The shaded area represents the range of RAPID3 scores that are ≤6. Therefore, the data points falling within the shaded area are discordant, ie patients not achieving MDA but having a RAPID3 score ≤6.

**Supplementary Figure 3 Correlation between disease activity in psoriatic arthritis in remission (DAPSA) scores and rapid assessment of patient index (RAPID3) scores across all individual patient visits.**

The scatter plot shows the distribution of DAPSA scores compared to the RAPID3 scores for individual patient visits across the entire study. The points that lie within the shaded area in the bottom left of the graph demonstrate individual patient visits in which patients who achieved DAPSA-LDA (DAPSA score ≤14) scored RAPID3 ≤10, the optimal cut point identified by ROC analysis for DAPSA-LDA. The points that lie within the shaded area in the top right of the graph demonstrate individual patient visits in which patients did not meet DAPSA-LDA and scored RAPID3 >10. All points lying outside the shaded square represent discordant results.
